# TMP-SMX-associated acute respiratory distress syndrome with DAIDE pathology in a Korean patient lacking HLA-B*07:02 and C*07:02: A case report

**DOI:** 10.1097/MD.0000000000046427

**Published:** 2025-12-12

**Authors:** Yooyoung Chong, Yong Chae Jung, Kyung-Hee Kim, Jae Hyeon Yu, Jun Wan Lee

**Affiliations:** a Department of Thoracic and Cardiovascular Surgery, Chungnam National University Hospital, Daejeon, Republic of Korea; b Department of Pathology, Chungnam National University, School of Medicine, Daejeon, Republic of Korea.

**Keywords:** acute respiratory distress syndrome, case report, extracorporeal membrane oxygenation, HLA typing, sulfamethoxazole drug combination, trimethoprim

## Abstract

**Rationale:**

Trimethoprim-sulfamethoxazole (TMP-SMX)–induced acute respiratory distress syndrome (ARDS) is a rare but potentially fatal adverse drug reaction, most commonly reported in young, otherwise healthy individuals. Although recent studies from the United States have identified a strong association with human leukocyte antigen (HLA)-B07:02 and C07:02 alleles, no cases have been reported in East Asian populations.

**Patient concerns:**

A 37-year-old Korean male with rheumatoid arthritis–associated interstitial lung disease developed rapidly progressive dyspnea and hypoxemia after receiving TMP-SMX for *Pneumocystis jirovecii* polymerase chain reaction positivity.

**Diagnoses:**

The patient was diagnosed with TMP-SMX–induced ARDS. Surgical lung biopsy demonstrated diffuse alveolar injury with delayed epithelialization. HLA genotyping confirmed the absence of HLA-B07:02 and C07:02 alleles.

**Interventions:**

TMP-SMX was discontinued, and the patient received maximal supportive therapy, including corticosteroids, mechanical ventilation, and venovenous extracorporeal membrane oxygenation.

**Outcomes:**

Despite aggressive management, the patient required prolonged extracorporeal membrane oxygenation support and was transferred for lung transplantation evaluation due to persistent severe respiratory failure.

**Lessons:**

This case represents the first reported TMP-SMX–induced ARDS with diffuse alveolar injury with delayed epithelialization pathology in an East Asian individual, suggesting ethnic variability in HLA-associated risk alleles. The findings underscore the need for population-specific pharmacogenomic investigation in severe drug-induced lung injury.

## 1. Introduction

Trimethoprim-sulfamethoxazole (TMP-SMX)-induced acute respiratory distress syndrome (ARDS) is a rare but potentially fatal adverse drug reaction, most reported in young, otherwise healthy individuals.^[1,2]^ Recent literature from the United States has identified a strong association with HLA-B*07:02 and C*07:02 alleles.^[1,3,4]^ TMP-SMX is a widely prescribed antibiotic, commonly used for prophylaxis and treatment of Pneumocystis jirovecii pneumonia, urinary tract infections, and other bacterial infections. In East Asia, TMP-SMX is frequently administered both in immuno-compromised populations and in routine clinical practice, reflecting its broad spectrum and cost-effectiveness. Studies from Japan have documented thousands of non-HIV immuno-compromised patients treated with TMP-SMX.^[5]^ Despite this high regional exposure, severe pulmonary adverse reactions, such as ARDS, remain uncommon but clinically significant, underscoring the importance of careful documentation to inform practice.^[6]^

Adverse drug reactions to TMP-SMX have been increasingly linked to host genetic variability, particularly human leukocyte antigen (HLA) alleles. Recent pharmacogenomic studies have demonstrated strong associations between specific HLA types and drug hypersensitivity syndromes, with significant inter-ethnic variability influencing risk profiles.^[7]^ Recent pharmacogenomics studies in Southeast and East Asia have identified multiple HLA alleles associated with severe cutaneous adverse drug reactions to co-trimoxazole and sulfamethoxazole/trimethoprim, including HLA-B*13:01, B*38:02, B*15:02, and C*08:01.^[8]^ One recent study also showed association of HLA-B*14:01 and B*35:01 with TMP-SMX–induced liver injury in an East Asian population.^[9]^ These findings reinforce population-specific HLA variability and support the importance of reporting novel TMP-SMX ARDS cases in East Asia. However, evidence regarding HLA associations with TMP-SMX–induced pulmonary injury remains limited, and their role in non-cutaneous manifestations such as ARDS has not been well established.

Histopathologically, TMP-SMX–induced ARDS has been associated with a rare but distinctive pattern termed drug-induced acute interstitial and diffuse alveolar damage (DAD) with delayed epothelialization.^[6,10]^ Diffuse alveolar injury with delayed epithelialization (DAIDE) is characterized by DAD accompanied by marked interstitial infiltration of eosinophils, a finding that helps differentiate drug-induced injury from infectious or autoimmune-related causes of ARDS. Clarifying and documenting this entity is crucial, as it provides pathologic evidence supporting drug causality in cases where clinical presentation and exposure history may be ambiguous.

Here, we report the first known case of TMP-SMX–associated ARDS with DAIDE pathology in an East Asian patient lacking HLA-B*07:02 and HLA-C*07:02, alleles previously reported in association with this adverse reaction. This case underscores the importance of integrating clinical, genetic, and histopathologic assessment in the evaluation of severe drug-induced lung injury (DLI).

## 2. Case description

A 37-year-old Korean man was transferred to our department for further management of a massive hemothorax, which developed following pleural catheter drainage of a right-sided pleural effusion. He had a medical history of rheumatoid arthritis-associated interstitial lung disease (RA-ILD), treated with low-dose corticosteroids (prednisolone 5 mg twice daily) and methotrexate (10 mg once daily) for the preceding 4 months. The patient had no history of smoking or occupational inhalation exposures and had no history of other potential culprits for DLI, including nonsteroidal anti-inflammatory drugs, biologic agents, or recent initiation of other disease-modifying antirheumatic drugs. There was no history of thoracic radiation therapy, and no clinical or radiologic evidence of infection-related lung injury was identified prior to TMP-SMX exposure.

**Figure 1.**
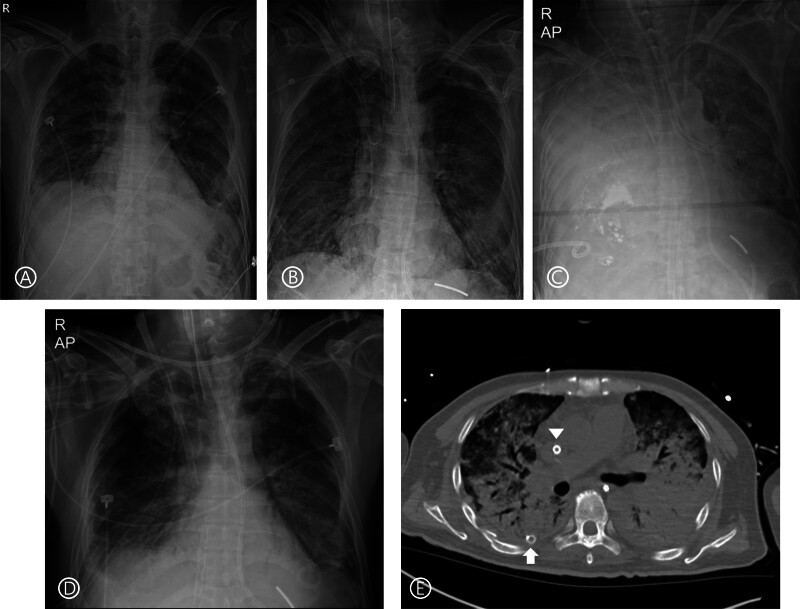
Serial chest radiographs and chest CT images illustrating the clinical course. (A) Initial chest radiograph at another hospital shows ill-defined peripheral ground-glass opacities and consolidations in both lungs, predominantly in the lower lobes, suggestive of pneumonia including interstitial lung disease. (B) On the eighth hospital day, diffuse dense consolidations are noted in both lungs, raising differential considerations of pulmonary edema, acute respiratory distress syndrome, and combined atypical pneumonia; a focal dense consolidation is present in the left lower lung zone. (C) On the thirteenth hospital day, pre-transfer chest CT scan demonstrates right pleural effusion after catheter drainage (arrow = trans-pleural catheter). (D) On the day of transfer, chest radiograph shows a massive right pleural effusion with bilateral pulmonary edema in the setting of V-V-ECMO placement. (E) On the fifth postoperative day, partial improvement of bilateral infiltrates is evident. (F) On the thirteenth postoperative day, contrast-enhanced CT reveals extensive bilateral air-space consolidation and ground-glass attenuation, with increased density in dependent regions consistent with DAD (arrow = right chest tube; arrowhead = ECMO inflow cannula in superior vena cava). CT = computed tomography, DAD = diffuse alveolar damage, V-V-ECMO = veno-venous extracorporeal membrane oxygenation.

Two weeks prior to transfer, he was admitted to another hospital with worsening dyspnea and high-grade fever, initially presumed to be an exacerbation of RA-ILD (Fig. 1A). He was treated with methylprednisolone pulse therapy (500mg daily) followed by 60mg once daily, along with intravenous cefepime (2 g 3 times daily), and supplemental oxygen therapy. Microbiological and viral testing was negative except for Pneumocystis Jiroveci PCR result from a bronchial aspiration sample obtained via bronchoscopy examination on the first hospital day. However, distinguishing between an acute exacerbation of RA-ILD, pneumocystis pneumonia, and DLI posed a significant diagnostic challenge during initial hospital course. The overlapping clinical and radiological features of these conditions complicated early decision-making, particularly in the context of immunosuppression and a positive Pneumocystis jirovecii PCR result. Intravenous TMP-SMX was initiated with therapeutic dose (TMP 160mg/SMX 800mg q12h) and continued for 15 days. After TMP-SMX discontinuation, corticosteroid therapy was continued according to a low-dose regimen (Meduri protocol: 1 mg/kg/day of methylprednisolone with gradual taper), reflecting standard critical care practice for ARDS management.

Despite medical treatment, he developed rapidly progressing respiratory failure, requiring endotracheal intubation and mechanical ventilation on the 8-hospital day (Fig. 1B). At the time of intubation, he was receiving high-flow nasal cannula oxygen (Optiflow, FiO₂ 0.9, 30 L/min), with arterial blood gas showing PaO₂ 71 mm Hg and SpO₂ 88%, corresponding to a PaO₂/FiO₂ ratio of 79, consistent with severe ARDS under the Berlin definition. According to outside hospital records, the patient was initially managed with conventional mechanical ventilation in controlled mandatory ventilation mode with FiO₂ 1.0, respiratory rate 22/min, pressure support 15 cmH₂O, PEEP 8 cmH₂O, and tidal volumes ranging from 360 to 480 mL (6–8 mL/kg predicted body weight). The plateau pressure at this stage was 22 cmH₂O. Immediately prior to veno-venous extracorporeal membrane oxygenation (V-V ECMO) initiation, the ventilator remained on controlled mandatory ventilation mode with FiO₂ 1.0, respiratory rate 22/min, pressure support increased to 20 cmH₂O, PEEP 10 cmH₂O, and tidal volumes decreased to 240 to 320 mL, with plateau pressure rising to 30 cmH₂O. At this point, his PaO₂ was 52 mm Hg, giving a PaO₂/FiO₂ ratio of 52, again confirming severe ARDS.

Due to refractory hypoxemia and rising plateau pressures, V-V ECMO was initiated with an initial flow of 3.2 L/min, sweep gas flow of 5 L/min, and FiO₂ of 1.0. After transfer to our hospital, his oxygenation on ECMO stabilized with PaO₂ maintained between 60 to 70 mm Hg. On the day of transfer, he also underwent trans-pleural catheter drainage for pleural effusion. A post-procedural chest computed tomography scan revealed a pleural effusion in the right hemithorax (Fig. 1C). Due to ongoing bloody drainage from the pleural catheter, the patient was transferred to our hospital for emergent surgical control of bleeding. On admission, he was hypotensive and tachycardic. A chest radiograph revealed a massive pleural effusion in the right hemithorax (Fig. 1D), and he subsequently underwent an emergent exploration via video-assisted thoracic surgery for traumatic hemothorax. The right lower lobe was injured by the pleural catheter, and hemostasis was achieved following thoracoscopic wedge resection.

Postoperatively, he was admitted to intensive care unit for ongoing mechanical ventilation and ECMO support. Bronchoalveolar lavage (BAL) culture revealed no growth of bacterial, mycobacterial, or fungal elements, and extended respiratory pathogen polymerase chain reaction was negative for respiratory viruses. Considering the rapidly progressing respiratory failure following recent exposure to a treatment dose of TMP-SMX, with no evidence of infectious etiologies in a medically controlled RA patient and considering previous case series, the diagnostic possibility of TMP-SMX-associated ARDS was raised. Although Pneumocystis jirovecii pneumonia (PCP) was initially suspected due to 1 positive PCR result from bronchial aspiration at the outside hospital, subsequent diagnostic studies – including repeat bronchial aspiration PCR, Gomori methenamine silver (GMS) staining of the resected lung specimen, and serial sputum cultures with GMS staining – were consistently negative, making PCP highly unlikely. Instead, histopathological examination of the resected lung demonstrated features of drug-associated indeterminate DAD with delayed epithelialization, characterized by DAD with focal eosinophilic infiltration. These findings supported the diagnosis of TMP-SMX–induced lung injury rather than infectious pneumonia.

Serial laboratory findings and clinical status are summarized in Table 1. White blood cell counts progressively increased, peaking at 13,350/µL at the time of transfer. C-reactive protein (CRP) initially declined after corticosteroid therapy but rose sharply during respiratory deterioration, exceeding 20 mg/dL postoperatively. Hepatic enzymes fluctuated without clear evidence of hepatotoxicity. Serum creatinine showed a transient increase to 1.87 mg/dL during ECMO initiation, consistent with acute kidney injury, but normalized thereafter. These laboratory and clinical features further supported an acute inflammatory, DLI process rather than persistent infection.

**Table 1:** Timeline table for hospital course.

Hospital day/event	Clinical status	Key investigations	Treatment/intervention
Day 0 (outside hospital admission)	Dyspnea, fever; presumed RA-ILD exacerbation	WBC 6700; CRP 6.2; PJP PCR (+) from bronchial aspiration	Methylprednisolone pulse 500 mg IV; cefepime 2 g TID; oxygen via nasal prong 4 L/min
Day 1–5	Partial improvement then worsening hypoxemia	CRP ↓ to 1.2; WBC 9040	Continued steroids (60 mg IV daily); started TMP-SMX IV (160/800 mg q12h); MTX discontinued
Day 8	Rapid respiratory failure	WBC 9780; CRP 10.5	Endotracheal intubation; CMV (FiO₂ 1.0, RR 22, PS 15, PEEP 8, TV 360–480 mL); PaO₂/FiO₂ ≈120
Day 9	Refractory hypoxemia	PaO₂ 52 on FiO₂ 1.0; WBC 12,590	V-V-ECMO initiated (flow 3.2 L/min, sweep 5 L/min, FiO₂ 1.0); steroids transitioned to Meduri protocol (low-dose IV methylprednisolone)
Day 11	Transferred to our hospital for hemothorax	Pleural effusion with bloody drainage	Trans-pleural catheter drainage; continued V-V-ECMO support
Day 12	Surgical control of bleeding	Chest CT: right hemothorax	Thoracotomy and hemostasis; BAL culture and PCR negative; pathology showed DAIDE with no PJP (GMS stain -)
Post-op (ICU)	Persistent severe ARDS on ECMO	PaO₂/FiO₂ <80; CRP ↑ to > 20; WBC 13,350	V-V-ECMO, lung-protective ventilation, prone positioning; no vitamin C or additional immunosuppressants attempted

ARDS = acute respiratory distress syndrome, BAL = bronchoalveolar lavage, CT = computed tomography, DAIDE = diffuse alveolar injury with delayed epithelialization, PJP = pneumocystis jirovecii pneumonia, RA-ILD = rheumatoid arthritis-associated interstitial lung disease, TMP-SMX = trimethoprim-sulfamethoxazole, V-V-ECMO = veno-venous extracorporeal membrane oxygenation.

**Figure 2.**
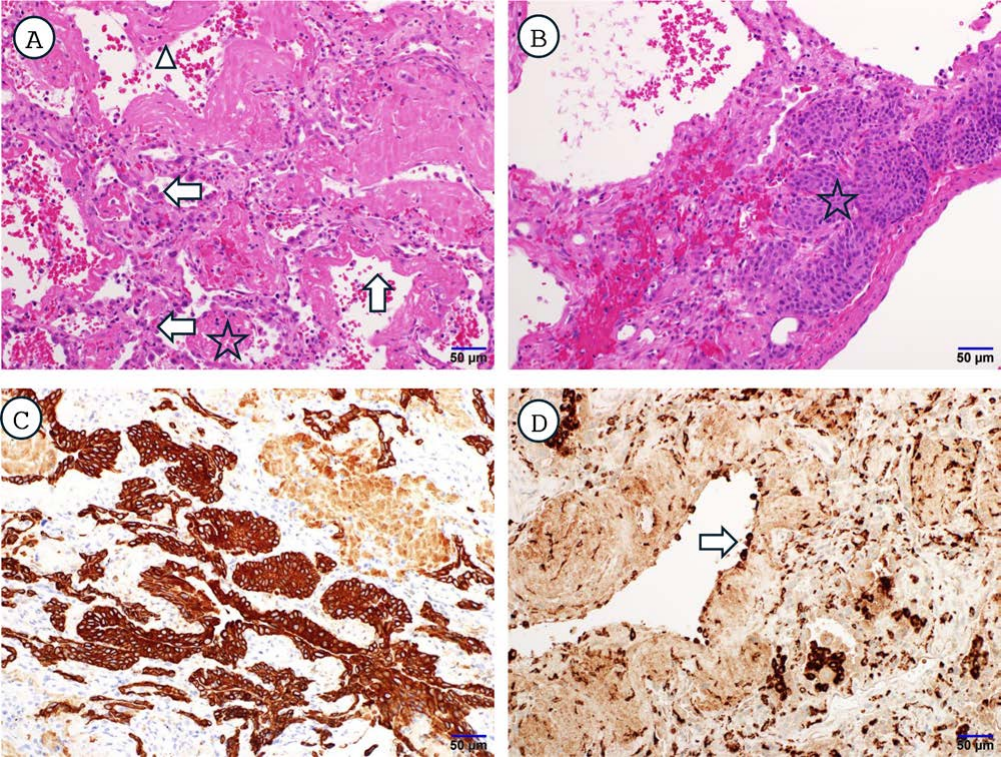
Histopathologic findings of lung biopsy confirming DAIDE. (A) Diffuse alveolar damage pattern with eosinophilic hyaline membranes (upward arrow), intra-alveolar proteinaceous exudate (asterisk), and pneumocyte hyperplasia (leftward arrow) (H&E, ×200). (B) Regenerative squamous cell proliferations with basaloid buds (asterisk) (H&E, ×200). (C) Pancytokeratin stain highlights peribronchiolar basaloid buds (CK AE1/AE3 immunohistochemistry, ×200). (D) CD68-positive macrophages are attached along alveolar walls (rightward arrow) (CD68 immunohistochemistry, ×200). Scale bars = 50 μm. Annotations: arrows indicate key morphologic features (see descriptions above). DAIDE = drug-associated interstitial damage with eosinophilia.

**Figure 3.**
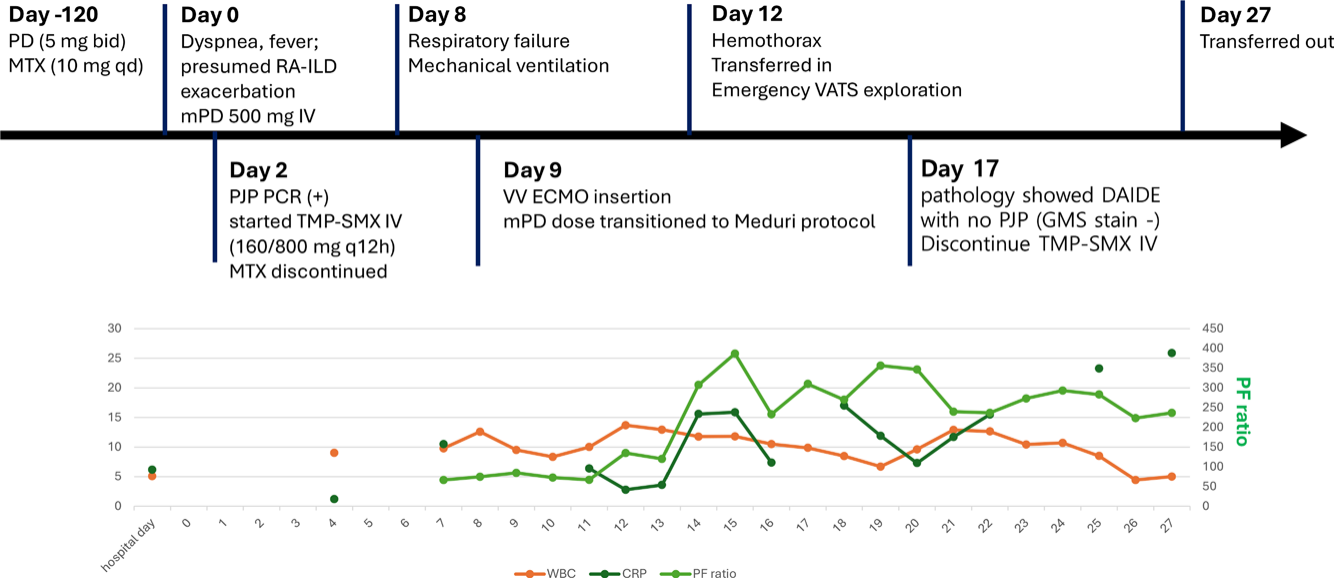
Summary of progression. Timeline diagram depicting clinical progression. A 37-yr-old male with RA-ILD presented with dyspnea and fever. Despite corticosteroid pulse therapy and TMP-SMX for confirmed PJP, respiratory failure worsened, requiring intubation and V-V-ECMO. Surgical hemostasis was performed for right hemothorax during the postoperative course. Changes in white blood cell count (WBC, cells/µL; orange line), C-reactive protein (CRP, mg/L; green line), and PaO₂/FiO₂ ratio (PF ratio, mm Hg; light green line) according to patient progress. CRP = C-reactive protein, DAIDE = diffuse alveolar injury with interstitial edema, mPD = methylprednisolone, MTX = methotrexate, PD = prednisolone, PF ratio = PaO₂/FiO₂ ratio, PJP = pneumocystis jirovecii pneumonia, RA-ILD = rheumatoid arthritis-associated interstitial lung disease, TMP-SMX = trimethoprim-sulfamethoxazole, V-V-ECMO = veno-venous extracorporeal membrane oxygenation, WBC = white blood cell.

Histopathologic examination of the resected lung specimen demonstrated DAD pattern, showing hyaline membrane along alveolar wall, intra-alveolar proteinaceous cellular exudate or solid fibrin deposits, and regenerative peribronchiolar basaloid pods. It showed a paucity of macrophages lining alveolar walls, but some macrophages attached on the denuded alveolar walls. These findings were consistent with DAIDE (Fig. 2). This pattern is characterized by widespread alveolar septal thickening with interstitial infiltration of eosinophils, prominent type II pneumocyte hyperplasia, and hyaline membrane formation. Unlike conventional DAD, where neutrophilic infiltration predominates, the presence of dense eosinophilic infiltration is highly suggestive of drug-associated lung injury and is considered a histopathologic hallmark of TMP-SMX–related ARDS in previously reported series. The diagnostic workflow and timeline of clinical events are summarized (Fig. 3).

Special stains including GMS and PCR panels for bacteria and viruses were negative. HLA typing was performed using a polymerase chain reaction–sequence-specific oligonucleotide probe (PCR-SSOP) method. This assay has been validated as a highly sensitive (>95%) and specific (>98%) technique for detecting known HLA alleles associated with adverse drug reactions. In this patient, the absence of HLA-B*07:02 and C*07:02 – previously implicated in TMP-SMX hypersensitivity – further underscored the novelty of this case in the East Asian population, where data remain scarce. HLA genotyping results are summarized in Table 2.^[11–21]^

**Table 2:** Patient HLA genotype and selected published clinical associations.

Locus	Allele	Known clinical associations (summary)	Refs
HLA-A	A*33:33	No established disease/drug association identified in major meta-analyses or guidelines.	–
HLA-A	A*33:03	Associations reported: (i) allopurinol‑induced SCAR (SJS/TEN/DRESS) in Asian cohorts; (ii) CM/acetaminophen‑associated SJS/TEN with severe ocular complications (phenotype‑specific).	[11–13]
HLA-B	B*44:03	Associated with cold‑medicine/acetaminophen‑related SJS/TEN with severe ocular complications in Japanese/Indian/Brazilian cohorts; non‑significant in a Korean cohort.	[14,15]
HLA-B	B*58:01	Strong, guideline‑level association with allopurinol‑induced SCAR (SJS/TEN/DRESS); screening recommended in high‑risk populations.	[15,16]
HLA-C	C*03:02	Reported association with allopurinol‑induced SCAR (including DRESS); linkage disequilibrium with B*58:01 in some populations.	[11,17]
HLA-C	C*14:02	Reported (weak/limited) association with phenytoin‑induced DRESS in a pediatric Thai cohort.	[18]
HLA-DRB1	DRB1*04:05	Established risk allele for rheumatoid arthritis in East Asians.	[19]
HLA-DRB1	DRB1*09:01	Associated with type 1 diabetes susceptibility in Asian populations.	[20]
HLA-DQB1	DQB1*03:01	Associated with bullous pemphigoid in large cohorts.	[21]
HLA-DQB1	DQB1*04:02	No consistently replicated disease/drug association identified.	–

HLA = human leukocyte antigen.

No drug re-challenge or drug allergy testing was attempted in this case, given the life-threatening presentation and the risk of recurrence. Clinical improvement after withdrawal of TMP-SMX, combined with the histopathologic and clinical findings, was considered sufficient for diagnosis. Thus, TMP-SMX–induced ARDS was determined to be the most likely diagnosis after exclusion of infectious and alternative noninfectious causes.

Upon recognition of TMP-SMX–associated ARDS, the drug was immediately discontinued. Corticosteroid therapy was continued with low-dose intravenous methylprednisolone protocol consistent with the Meduri regimen, aimed at modulating persistent lung inflammation in ARDS. The patient also received lung-protective mechanical ventilation, V-V ECMO, and adjunctive prone positioning. No experimental adjuvant therapies such as high-dose vitamin C or additional immunosuppressants were attempted. Methotrexate, previously administered for rheumatoid arthritis, was discontinued upon admission to minimize potential contribution to lung injury. Despite these measures, respiratory failure persisted, complicated by hemothorax requiring surgical intervention.

Despite maximal medical therapy including lung-protective ventilation (ultra-low tidal volume), paralytics, recruitment maneuvers, positive end-expiratory pressure titration, pulmonary vasodilators and intravenous corticosteroids continued to require ECMO support without signs of readiness for weaning. A chest radiograph on fifth postoperative day showed partial improvement of bilateral infiltrates on both lung fields under V-V ECMO support (Fig. 1E). Throughout the next 2 weeks, his condition fluctuated without any significant improvement. A computed tomography scan of the chest on the thirteenth postoperative day revealed bilateral lower zone predominant ground-glass opacity and air-space consolidation, typical of DAD (Fig. 1F). In the setting of an inability to further wean respiratory supports, he was considered for lung transplantation and eventually, transferred to another institution under V-V ECMO support. Unfortunately, he remained on ECMO support while awaiting transplantation and died during the waiting period. Therefore, no further long-term follow-up data was available.

## 3. Discussion

Drug-induced lung injury (DLI) represents a critical challenge, particularly in patients with underlying autoimmune disease and immunosuppressive therapy. In this case, the differential diagnosis included methotrexate-induced lung injury (MTX-DLI), exacerbation of rheumatoid arthritis-associated interstitial lung disease (RA-ILD), and TMP-SMX–induced ARDS. MTX-DLI often presents with subacute symptoms and ground-glass opacities, typically improving with drug discontinuation and corticosteroids. RA-ILD exacerbation is characterized by DAD in the absence of new drug exposure.^[22–24]^ However, the temporal relationship with TMP-SMX initiation, rapid progression of respiratory failure, absence of infectious etiologies, and histopathologic confirmation of diffuse alveolar injury with delayed epithelialization (DAIDE) supported TMP-SMX–induced ARDS as the most likely diagnosis.

DAIDE is a rarely described histopathologic finding in DLI, distinct from the classic DAD pattern.^[6,10]^ Its presence in this case underscores the unique pathological spectrum associated with TMP-SMX-induced ARDS. A small number of global reports have described TMP-SMX–associated ARDS with histopathologic correlation, but to our knowledge, this is the first reported case with DAIDE in the East Asian population.^[10,24]^

The identification of HLA-B*58:01 in this patient provides novel insight into pharmacogenomic susceptibility. While B*58:01 is best known for its association with allopurinol-induced severe cutaneous adverse reactions, its implication in pulmonary toxicity raises the possibility of shared antigen presentation pathways.^[16,25]^ This finding highlights the need for larger pharmacogenomic studies to explore whether certain HLA alleles predispose to pulmonary hypersensitivity reactions to TMP-SMX.

Recent reviews on pharmacogenomics and HLA diversity in drug reactions emphasize that variability in HLA alleles contributes significantly to inter-individual susceptibility to severe adverse drug reactions, including immune-mediated organ toxicities.^[26–29]^ Allele-restricted immune responses, mediated by drug–HLA–T cell receptor interactions, provide mechanistic insight into how genetic background drives tissue-specific injury. These reviews also stress that allele frequencies differ substantially between ethnic groups, underscoring the importance of region-specific pharmacogenomic studies. In this context, the identification of HLA-B*58:01 in our patient with TMP-SMX–associated ARDS raises the possibility that alleles beyond the well-established B*07:02 and C*07:02 may contribute to pulmonary hypersensitivity. Incorporating pharmacogenomic screening into clinical decision-making may eventually help identify high-risk individuals before exposure to potentially life-threatening medications may eventually help identify high-risk individuals before exposure to potentially life-threatening medications.

Management of TMP-SMX–induced ARDS remains largely supportive. In our case, despite maximal protective mechanical ventilation, the patient developed refractory hypoxemia necessitating V-V ECMO. ECMO provided a bridge to potential lung recovery and lung transplantation evaluation. However, the patient’s failure to wean and eventual death while awaiting transplantation underscore both the potential and limitations of ECMO in TMP-SMX–associated ARDS.

This case has important limitations. First, causality cannot be confirmed definitively due to the absence of drug re-challenge, which would have been ethically unsafe. Second, HLA testing was limited to selected alleles using PCR-SSOP rather than broader sequencing. Third, long-term follow-up was unavailable because the patient died while awaiting lung transplantation. Nonetheless, this case expands the clinicopathologic spectrum of TMP-SMX–associated ARDS, highlights the importance of DAIDE recognition, and raises potential pharmacogenomic associations relevant to East Asian populations.

## Author contributions

**Conceptualization:** Yooyoung Chong, Jae Hyeon Yu, Jun Wan Lee.

**Data curation:** Yooyoung Chong, Yong Chae Jung.

**Investigation:** Yong Chae Jung.

**Project administration:** Yooyoung Chong, Yong Chae Jung, Jae Hyeon Yu.

**Supervision:** Jae Hyeon Yu.

**Validation:** Kyung-Hee Kim.

**Visualization:** Kyung-Hee Kim.

**Writing – original draft:** Yooyoung Chong, Kyung-Hee Kim.

**Writing – review & editing:** Yooyoung Chong, Jun Wan Lee.
